# Higher Intensity Quality Improvement Campaign Yields Greater Reduction in Medical Glove Use: The “Gloves Off!” Campaign

**DOI:** 10.5811/westjem.56980

**Published:** 2026-09-24

**Authors:** Lai Heng Foong, Matthew Knox, Arunima Malik, Wendy Hird

**Affiliations:** * South Western Sydney Local Health District, Bankstown Lidcombe Hospital, Emergency Department, Bankstown, New South Wales, Australia; † University of Sydney, Heat and Health Research Centre, Sydney, New South Wales, Australia; ‡ Central Coast Local Health District, Wyong Hospital, Emergency Department, Wyong, New South Wales, Australia; § University of Sydney, School of Physics, Integrated Sustainability Analysis, Sydney, New South Wales, Australia; || University of Sydney, Business School, School of Accounting, Governance and Regulation, Sydney, New South Wales, Australia; # South Western Sydney Local Health District, Sustainability Department, Liverpool, New South Wales, Australia

## Abstract

**Introduction:**

The healthcare sector contributes 5.4–7.0% of Australia’s total carbon dioxide (CO2) emissions. Among the most ubiquitous items used in healthcare organisations, whether it be hospital, clinic, or community health centres, are gloves.

**Methods:**

We reviewed two case studies of the “Gloves Off!” sustainability initiative to compare reduction in glove use and improvement in hand hygiene practice—one that featured a “low-intensity” campaign (Hospital A) and the other a “high-intensity” campaign (Hospital B).

**Results:**

A comparison of glove use over the year before and after implementation of the Gloves Off! campaign shows that Hospital A achieved a 2.6% reduction (0.4% reduction per patient), while Hospital B achieved an 11.4% reduction in total glove consumption (7.8% reduction in glove use per patient).

**Conclusion:**

Sustainability initiatives can be initiated within busy emergency departments with reduced unnecessary use of nonsterile gloves, decreased CO2 emissions, and cost savings.

## INTRODUCTION

The healthcare sector contributes between 5.4–7.0% of Australia’s total carbon dioxide (CO_2_) emissions.^1^, ^2^ Among the most ubiquitous items used in healthcare organisations, whether it be hospital, clinic or community health centres, are nonsterile gloves (NSG). Globally, gloves are used by clinicians for infection control and personal protection. Many reports, including from the World Health Organization (WHO), have indicated that NSG are being used inappropriately.^6^ Unnecessary glove use wastes money and increases medical waste, contributing to CO_2_ emissions from the healthcare sector. In addition, inappropriate glove use has been demonstrated to worsen hand hygiene rates.^6^ The WHO World Hand Hygiene Day 2025 focused on proper use of disposable gloves during patient care.

Annually, the Hunter New England Local Health District in New South Wales, Australia, uses 30 million NSG at a cost of 1.5 million Australian dollars (AUD) and an estimated carbon footprint of one million kilograms of CO_2_ equivalent (kg CO_2_ eq). The John Hunter Hospital in Newcastle had a Net Zero clinical lead that first launched the *Gloves Off!* campaign in New South Wales in 2025. Their published study demonstrated^3^ a reduction after the campaign was initiated.

In a study in the United Kingdom, NSG contributed to 45% of the carbon footprint of personal protective equipment used in healthcare.^4^ An average of 500 million gloves were sent for use in the National Health Service (NHS) and social care per month in 2023, which translates to 16.7 million gloves per day.

Studies have shown that the unnecessary use of gloves is as high as 52.9% of care episodes^5^ to 99% of low-risk procedures.^6^ The “*Gloves Off!”* campaign has been launched in multiple hospitals in Australia and globally. Busy clinicians are often neither able nor motivated to embark on a quality improvement (QI) journey due to competing clinical demands and lack of dedicated paid time for QI projects.

### Objective

The objective of this study was to evaluate the effectiveness of a high-intensity versus low-intensity staff awareness and culture-change approach within the *Gloves Off!* project, specifically in reducing unnecessary glove use in the emergency department (ED). We examined two case studies: one low-intensity and one high-intensity QI program. We focused on two New South Wales Health hospital EDs, one regional and the other urban metropolitan, with different time constraints and resources.

### Hospital A

Hospital A is an urban metropolitan facility in Sydney that sees approximately 60,000 presentations.

### Hospital B

Hospital B is a regional hospital one hour from Sydney that sees 75,000 presentations/year.

## METHODS

Our primary outcome measure was the reduction of glove use in the ED as evaluated by procurement data. We used procurement data for gloves as a surrogate measure of glove use. These data were obtained thorough a standardised database provided by our district sustainability manager.

The intervention included education about the proper use of gloves, presentation by an infection control nurse, and clinical champions. We compared two cases: a low-intensity model (Hospital A) and a high-intensity model (Hospital B). Using case studies from these two hospitals in separate local health districts in New South Wales, we assessed the roll out of the *Gloves Off!* campaign. We selected this approach to determine the workforce, time, and financial resources required to achieve appropriate glove-use behaviour and quantify the associated cost savings and CO_2_ reductions for each model. We compared total glove use over a one-year period before and after the intervention to evaluate the impact of the campaign to reduce glove use.

For Hospital A, which commenced the project in October 2024, we defined October 2023–September 2024 as the preintervention period and October 2024–September 2025 as the postintervention period. For Hospital B, which commenced the campaign in February 2024, we defined March 2023–February 2024 as the preintervention period and March 2024–February 2025 as the postintervention period. A full-year timeframe was selected to account for seasonal fluctuations in glove use—recognising that certain months exhibit predictable peaks or troughs—and to ensure a consistent and comparable measure of change across sites.

In addition, the evaluation considered two implementation-intensity models: a low-intensity model at Hospital A that featured an education campaign via email and in person; and a high-intensity model at Hospital B characterised by more comprehensive training and intervention activities, complemented by engagement activities. This allowed us to assess how differences in implementation intensity influenced changes in glove use.

### Hospital A: Low-Intensity Model

The ED of Hospital A established a sustainability working group just prior to the launch of the *Gloves Off!* project in 2024. The members then organised an education campaign to talk about the programme among nurses during usual in-services and doctors during teaching days (only one off). We used a presentation that had been produced by the New South Wales Net Zero Unit in education sessions for nurses and doctors. We included allied health staff and ward orderlies opportunistically. There was no formal launch but rather an announcement in our Microsoft Teams chat and via email. No additional funding was spent for the *Gloves Off!* campaign beyond coordination and utilising existing education sessions that had already been planned. Emergency department sustainability champions within the working group educated clinical staff about appropriate glove use and displayed reminder posters.

### Hospital B: High Intensity Model

The *Gloves Off!* programme was implemented in the Hospital B ED using a multifaceted QI approach. Baseline data collection included direct observation of hand hygiene and glove-use behaviours by ED staff, along with analysis of departmental procurement data for nonsterile gloves. These data informed the development of a targeted education intervention.

A tailored educational package was delivered through multiple formats, including the following: interactive quizzes during junior medical officer orientation; nursing and clinician huddles; in-service sessions; and an on-site launch-day information stall. Additional reinforcement was provided through email communication and the ED newsletter. Supporting signage was installed in key departmental locations, such as glove dispensers and handwashing stations, and near clinical huddle boards. Post-launch evaluation included direct observation of hand hygiene and glove compliance alongside the collection of NSG) procurement data.

Campaign implementation at Hospital B cost approximately 600 AUD, which funded launch-day educational snacks for staff and to cover printing costs for department signage. Additionally, staff members volunteered a significant amount of unpaid time to complete the project.

## RESULTS

The results include a pre/postintervention comparison of glove use, both in absolute terms and in the number of gloves used per patient.

### Hospital A

At baseline, glove use was higher at Hospital A. Prior to the Gloves Off! campaign, the Hospital A ED used 1,310,180 gloves annually. Post-campaign, yearly glove use fell to 1,276,750—a reduction of 33,430 gloves (2.6% overall; 0.4% per patient), which is a carbon-equivalent to mitigating 1,136.3 kg CO_2_ eq.

### Hospital B

Before the *Gloves Off!* campaign was launched, the ED in Hospital B used 1,030,800 gloves annually. Post-campaign, glove use fell to 913,400, a reduction of 117,400 gloves (11.4% overall; 7.8% per patient), which equates to approximately 4,000 kg CO_2_ eq—equivalent to driving 11,000 km in a petrol-powered passenger vehicle.

Results of a comparison of glove use over the year before and after implementation of the campaign show that Hospital A achieved a 2.6% reduction in total glove consumption (0.4% reduction in glove use per patient), while Hospital B achieved an 11.4% reduction (7.8% reduction per patient). These findings suggest that both interventions were effective in reducing glove use, with the higher intensity approach implemented at Hospital B appearing to yield a greater impact. The analysis also highlights the potential influence of intervention intensity and implementation strategies on behavioural change in clinical settings. The education campaign was periodically reinforced to ensure that new and rotating staff were reached, including through orientation sessions and short educational videos distributed via the same communication channels.

In Hospital B, the project was promoted more broadly within the local health district through the intranet, email updates, sustainability forums, and a leadership forum. External dissemination occurred via the Australasian College for Emergency Medicine Sustainable Emergency Medicine Climate Advocacy Network. The project also received coverage through national media.

## DISCUSSION

Nonsterile gloves are an essential part of a healthcare worker’s equipment to prevent the spread of nosocomial infections and to ensure patient and staff safety. However, many studies have shown that NSG are used inappropriately in many healthcare settings.^7^,^8^ Furthermore, studies have shown that the use of NSG is associated with decreased hand hygiene compliance, increased risk of cross-contamination, and transmission of healthcare-associated infections.^9^,^10^

Our results from the two case studies show a difference in the reduction in NSG use between the the two hospitals. Reasons for this difference are multifactorial. Education alone cannot sustain the behavioural change required to maintain this shift in practice. According to the transtheoretical model of change, behaviour change involves many steps and even more to maintain it.^11^ The stages are precontemplation, contemplation, determination, action, relapse, and maintenance. (See Figure 2)

In a busy ED, most staff are focused on delivering patient care. It is difficult to add one more sustainability task in their daily workflow. The action-relapse-maintenance part of the cycle is the most difficult for busy clinicians to maintain. As healthcare professionals, we take an oath to “do no harm.” Unnecessary glove use generates emissions that contribute to climate change, thereby harming patients. We as clinicians have a responsibility to reduce this unnecessary harm. This simple *Gloves Off!* project allows us to decrease healthcare’s harm on the environment, improve patient care, and fulfill a mandatory job requirement (the QI initiative)—a triple win.

For Hospital B, the *Gloves Off!* project also delivered benefits beyond cost and environmental gains. It is a manageable initiative for busy clinicians that builds practical change-management skills and increases awareness of environmental impacts in the ED. The campaign at Hospital B’s ED catalysed the creation of an active sustainability team that has since delivered multiple high-impact projects and helped establish funded positions with protected time for resource-stewardship and clinical-care improvement work.

The annual cost savings associated with these two QI projects amounted to 1,009 AUD at Hospital A and 4,500 AUD at Hospital B, minus the 600 AUD it cost to implement the projects. The advantage of this project is that no additional expenses were required to conduct these QI projects, while encouraging clinicians to commit to a QI initiative that both saves money and benefits the environment.

## LIMITATIONS

Because both studies were conducted in a region where New South Wales Health has a Net Zero Unit, the results might not be generalizable to other states.

## CONCLUSION

Our two case studies using the *“Gloves Off!”* campaign demonstrate that we can easily introduce a sustainability initiative within busy EDs, with reduced use of unnecessary nonsterile gloves, lower CO_2_ emissions, and increased cost savings. Our comparison of two different “intensities” of intervention, with differing results, demonstrates that a higher intensity program leads to greater reductions and collateral benefits, including better engagement from clinicians and hospital executives, with improved sustainability for EDs. This scalable initiative could be expanded to other EDs in Australia and around the world.

## Figures and Tables

**Figure 1:**
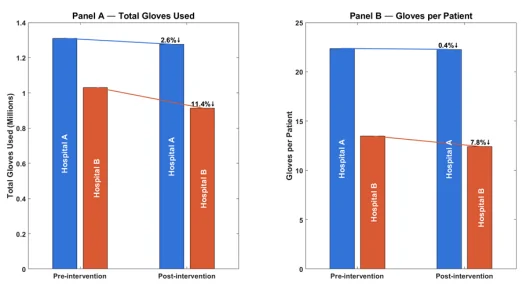
Comparison of glove use in Hospital A (low-intensity model) and Hospital B (high-intensity model), in absolute terms (Panel A) and gloves per presenting patient (Panel B), in a review of the effect of two quality improvement initiatives to reduce unnecessary use of nonsterile gloves in the emergency department

**Figure 2:**
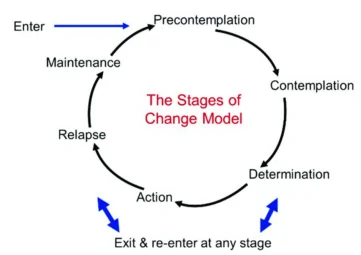
Stages of change model (Prochaska and De Clementi).^11^
